# New Medical Journals

**Published:** 1850-11

**Authors:** 


*New Medical Journals.*—The following two new Medical periodicals have recently been commenced:—

*The Western Medico-Chirurgical Journal;* Edited by I. F. Sanford, M. D., and Samuel G. Armor, M. D., Professors in the Iowa State University. This is a monthly of 32 pages. The terms are *two dollars per annum, in advance.*

*The New-York Register of Medicine and Pharmacy;* Edited by C. D. Griswold, M. D. Published semi-monthly, each number containing 16 pages. Terms, *one dollar per annum, in advance.*

We welcome these new candidates for favor, and tender to the Editors our best wishes.

